# Effects of melatonin and its receptor antagonist on retinal pigment epithelial cells against hydrogen peroxide damage

**Published:** 2012-06-20

**Authors:** Richard B. Rosen, Dan-Ning Hu, Min Chen, Steven A. McCormick, Joseph Walsh, Joan E. Roberts

**Affiliations:** 1 Department of Ophthalmology, New York Eye and Ear Infirmary, New York Medical College, New York, NY; 2 Tissue Culture Center, Department of Pathology and Laboratory Medicine, New York Eye and Ear Infirmary, New York Medical College, New York, NY; 3 Fordham University, New York, NY

## Abstract

**Purpose:**

Recently, we reported finding that circulating melatonin levels in age-related macular degeneration patients were significantly lower than those in age-matched controls. The purpose of this study was to investigate the hypothesis that melatonin deficiency may play a role in the oxidative damage of the retinal pigment epithelium (RPE) by testing the protective effect of melatonin and its receptor antagonist on RPE cells exposed to H_2_O_2_ damage.

**Methods:**

Cultured human RPE cells were subjected to oxidative stress induced by 0.5 mM H_2_O_2_. Cell viability was measured using the microculture tetrazoline test (MTT) assay. Cells were pretreated with or without melatonin for 24 h. Luzindole (50 μM), a melatonin membrane-receptor antagonist, was added to the culture 1 h before melatonin to distinguish direct antioxidant effects from indirect receptor-dependent effects. All tests were performed in triplicate.

**Results:**

H_2_O_2_ at 0.5 mM decreased cell viability to 20% of control levels. Melatonin showed dose-dependent protective effects on RPE cells against H_2_O_2_. Cell viability of RPE cells pretreated with 10^−10^, 10^−8^, 10^−6^, and 10^−4^ M melatonin for 24 h was 130%, 160%, 187%, and 230% of cells treated with H_2_O_2_ alone (all p<0.05). Using cells cultured without H_2_O_2_ as the control, cell viability of cells treated with H_2_O_2_ after pretreatment with 10^−10^-10^−4^ M melatonin was still significantly lower than that of the controls, suggesting that melatonin significantly decreased but did not completely abolish the in vitro cytotoxic effects of H_2_O_2_. Luzindole completely blocked melatonin’s protective effects at low concentrations of melatonin (10^−10^-10^−8^ M) but not at high concentrations (10^−6^-10^−4^ M).

**Conclusions:**

Melatonin has a partial protective effect on RPE cells against H_2_O_2_ damage across a wide range of concentrations (10^−10^-10^−4^ M). This protective effect occurs through the activation of melatonin membrane receptors at low concentrations (10^−10^-10^−8^ M) and through both the direct antioxidant and indirect receptor activation effects at high concentrations (10^−6^-10^−4^ M).

## Introduction

Age-related macular degeneration (AMD) is the leading cause of blindness in aged people in developed countries. The prevalence of AMD in Americans 40 years of age or older is 1.5%, and it has been estimated that 1.75 million people suffer from this disease in the United States [1].

The retinal pigment epithelium is a single layer of pigmented cells that have multiple functions essential to the maintenance of the overlying photoreceptors and hence to visual function. Oxidative stress has been implicated in the pathogenesis of AMD, possibly due to the detrimental effects of reactive oxygen species (ROS) on the retinal pigment epithelial (RPE) cells [2-6]. In support of this hypothesis, supplementation with antioxidants and zinc has been demonstrated in several studies to slow the progression of disease and preserve vision [6,7].

Recently, we found that the amount of 6-sulphatoxymelatonin (aMT6s) in nocturnal urine (a well established and reliable parameter for estimating peak circulating melatonin levels) in AMD patients was significantly lower than that of age- and gender-matched controls [8]. Whether reduced melatonin levels in AMD patients play a role in the occurrence of the disease remains unknown. It is therefore important to study the protective effects of melatonin on RPE cells against oxidative stress to determine the relationship between melatonin deficiency and RPE cell damage. A demonstration of RPE protection by melatonin would support the hypothesis that melatonin supplementation may be helpful for the prevention and treatment of AMD.

Furthermore, Baba et al. [9] reported that MT1 receptor transcripts were localized in mouse photoreceptor cells and in some inner retinal neurons. A diurnal rhythm in the dark-adapted electroretinography (ERG) responses was observed in wild-type (WT) mice, but not in melatonin membrane receptor 1 (MT1) receptor-deficient mice [MT1(−/−) mice]. Injection of melatonin during the day influenced ERG in WT mice but not MT1(−/−) mice. MT1(−/−) mice showed a significant decrease of photoreceptor nuclei and ganglion cells, compared with WT mice. These results demonstrate the functional significance of melatonin and MT1 receptors in the mammalian retina and create the basis for future studies on the therapeutic use of melatonin in retinal degeneration.

The detrimental effects of H_2_O_2_ on various cell types could be reduced by melatonin in the lymphoma cells [10], astrocytes [11], breast cancer cells [12], cerebellar granular neurons [13], pituitary cells [14,15], brain astrocytes [16], neuroblastoma cells [17], astroglial cells [18], spermatozoa [19], hepatoma cells [20] and motoneurons [21]. The mechanism of melatonin mediated cytoprotection has been documented as a direct antioxidant effect [12], or an indirect effect via the activation of melatonin receptors and relevant signal pathways [10,14,21], or through both direct and indirect effects [16,17], therefore, the melatonin protective effects and its mechanisms are highly cell type-specific. Very little is known about melatonin’s ability to protect RPE cells from H_2_O_2_ damage [22]. In this study, we looked at the influence of the dose and timing of melatonin administration on melatonin’s ability to protect cultured human RPE cells against H_2_O_2_-induced damage. In addition, the cell cultures were challenged, with and without the addition of luzindole, to determine the direct antioxidant versus indirect receptor-mediated effects of melatonin across a wide spectrum of concentrations.

## Methods

### Cell culture

The human RPE cell line, ARPE-19 (an immortal cell line from a 19-year-old donor), was obtained from American Type Culture Collections (Manassas, VA). Cells were cultured in Dulbecco’s Modified Eagle’s Medium (DMEM; Gibco, Carlsbad, CA), supplemented with 10% fetal bovine serum (FBS; Gibco). Cells were incubated in a humidified 5% CO_2_ atmosphere at 37 °C. After reaching confluence, cells were detached by trypsin-EDTA solution (Gibco), diluted 1:3–1:4, plated for subculture, and passaged routinely at a dilution of 1:3–1:4 every 5–7 days.

A new separate culture of primary human RPE cells was isolated from a donor eye (56 years old) and cultured as previously described [23,24]. Briefly, the anterior segment of the eye, the vitreous and the retina were excised. The RPE layer was immersed with trypsin-EDTA solution at 37 °C for 1 h. Culture medium with 10% FBS was added, and the RPE cells were isolated and collected under direct observation using a dissecting microscope. Isolated cells were centrifuged, re-suspended and seeded to a culture flask. Cells were also cultured in DMEM with 10% FBS. After reaching confluence, cells were subcultured as described above. Phase-contrast microscopy revealed pigmentation of RPE cells during the primary culture and the first and second subcultures. Cells displayed characteristic epithelial morphology throughout the culture period. The purity of the cell lines was demonstrated by immunocytochemical methods. RPE cells display positive staining of cytokeratin, whereas fibroblasts and melanocytes do not [25].

### Effects of H_2_O_2_ on the viability of retinal pigment epithelium cells

The effects of H_2_O_2_ on the viability of RPE cells were studied with the microculture tetrazoline test (MTT) test as described previously [24]. Briefly, RPE cells were plated in 96-well plates at a density of 5 × 10^3^ cells per well. After incubation for 24 h, H_2_O_2_ (Sigma, St. Louis, MO) was added to the wells at various final concentrations (0.05 mM, 0.1 mM, 0.25 mM, 0.5 mM, 0.75 mM, and 1.0 mM) and cultured for 24 h. Next, 50 µl of tetrazolium bromide, 3-(4,5-dimethylthiazol-2-yl)-2,5-diphenyltetrazolium bromide (1 mg/ml; Sigma), was added to each well and incubated for 4 h. The medium was withdrawn and 100 µl of DMSO (Sigma) was added to each well. The optical density was read at 540 nm using a microplate reader (Multiskan EX; Thermo, Vantaa, Finland). Cells cultured without H_2_O_2_ served as the controls. The effects of H_2_O_2_ on the viability of RPE cells were studied in the ARPE-19 cell line and the primary-culture RPE cell line separately. All groups were tested in triplicate.

### Effect of melatonin on H_2_O_2_-induced damage in retinal pigment epithelium cells

RPE cells were seeded as detailed above. Twenty-four hours later, melatonin (Sigma) was added to the culture medium at different concentrations (10^−10^ M to 10^−4^ M). After 1 h, 24 h, and 48 h, H_2_O_2_ was added to the medium at a final concentration of 0.5 mM and cultured for 24 h. Then, cell viability was evaluated by the MTT test as described above. Cells cultured with H_2_O_2_ but without melatonin were used as positive controls. This study was performed in the ARPE-19 cell line and the primary-culture RPE cell line separately. All groups were tested in triplicate.

### Influence of luzindole on the effects of melatonin on H_2_O_2_-induced damage in retinal pigment epithelium cells

RPE cells were seeded as previously detailed. After 24 h, luzindole (Sigma) was added to the culture medium at a final concentration of 50 μM. One hour later, melatonin was added to the culture medium at different concentrations (10^−10^ M to 10^−4^ M). After 24 h cultivation, H_2_O_2_ was added for a final concentration of 0.5 mM and cultured for 24 h. Then, cell viability was evaluated by the MTT test described above. This study was performed in the ARPE-19 cell line and the primary-culture RPE cell line separately. All groups were tested in triplicate.

### Statistical analysis

Statistical significances of differences in means throughout this study were calculated by an ANOVA one-way test in comparing data from more than two groups, and by the Student’s *t*-test in comparing data between two groups. A difference at p<0.05 was considered statistically significant.

## Results

### Cytotoxic effects of hydrogen peroxide on cultured human retinal pigment epithelium

**Figure 1:**
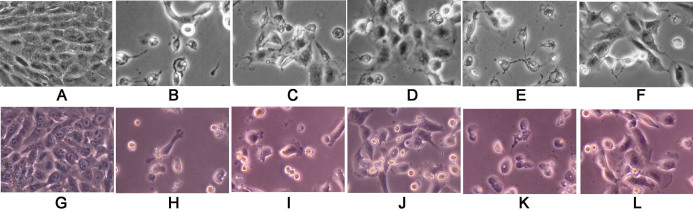
Melatonin protected the retinal pigment epithelium (RPE) cells against H_2_O_2_ damage, especially in high concentrations (10^−4^ M) and luzindole decreased the protective effects of melatonin. Phase-contrast microscopic images of the effects of melatonin and its membrane-receptor antagonist (luzindole) on retinal pigment epithelial cells against H_2_O_2_ damage. **A**-**F**: The RPE cells are from the ARPE-19 cell line (an immortal RPE cell line from a 19-year-old donor). **G**-**L**: The RPE cells are from the primary culture (PC) from the donor eye. ARPE-19 and PC cells were cultured with or without H_2_O_2_, melatonin, and luzindole, as follows: without H_2_O_2_, melatonin, and luzindole(**A** and **G**); with H_2_O_2_ at 0.5 mM concentrations for 24 h (**B** and **H**); with H_2_O_2_ and a pretreatment of melatonin at 10^−10^ M (**C** and **I**) or at 10^−4^ M (**D** and **J**) for 24 h; or with luzindole (50 μM, 1 h), followed by melatonin at 10^−10^ M (**E** and **K**) or at 10^−4^ M for 24 h (**F** and **L**). H_2_O_2_ was then added and the ARPE-19 and PC cells cultured for 24 h.

**Figure 2:**
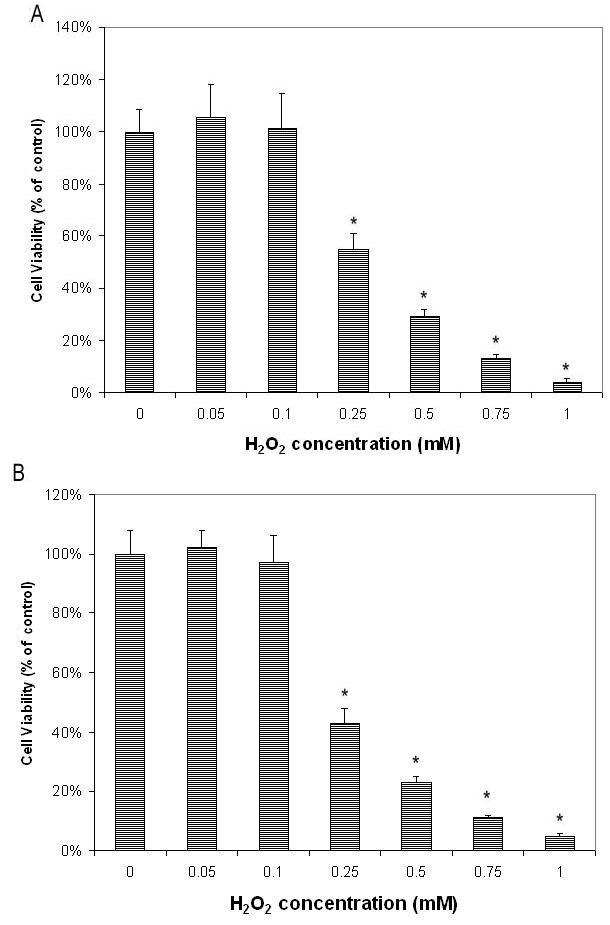
H_2_O_2_ dose-dependently decreased cell viability of retinal pigment epithelial cells as tested by microculture tetrazoline test. Retinal pigment epithelial (RPE) cells were plated in 96-well plates and treated with various concentrations of H_2_O_2_ for 24 h. Cell viability was evaluated by the microculture tetrazoline test (MTT) test and expressed as percentage of that in cells without H_2_O_2_ (mean±standard deviation [SD] in triplicate tests). **A**: H_2_O_2_ showed dose-dependent cytotoxic effects on ARPE-19 cells (cells from an immortal RPE cell line from a 19-year-old donor) at concentrations from 0.25 mM to 1.00 mM (p<0.05, compared to cells cultured without H_2_O_2_. **B**: Studies of primary cultures of human RPE cells isolated from donor eye showed similar results. *p<0.05, compared with the controls (cells cultured with H_2_O_2_ alone).

H_2_O_2_ showed dose-dependent cytotoxic effects on cultured human RPE cells (ARPE-19) at concentrations from 0.25 mM to 1.00 mM (Figure 1 and Figure 2). Cell viability in cells treated with 0.5 mM H_2_O_2_ decreased by 71%, compared to the controls (cells not treated with H_2_O_2_). Therefore, 0.5 mM H_2_O_2_ was selected as the concentration used for subsequent experiments. H_2_O_2_ had a similar cytotoxic effect on the primary-culture human RPE cells (Figure 2B).

### Ability of melatonin to protect retinal pigment epithelium cells against H_2_O_2_ damage

**Figure 3:**
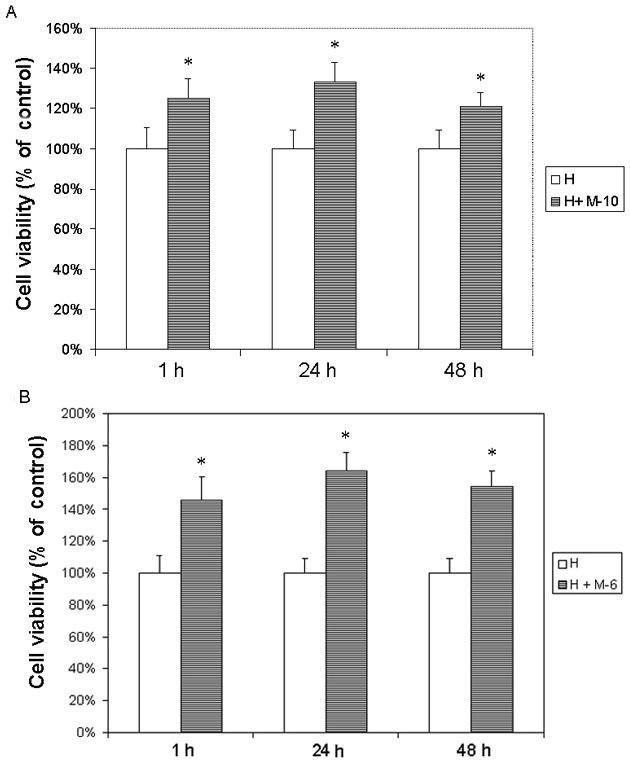
The ARPE19 cells (an immortal retinal pigment epithelial cell line from a 19-year-old donor) were treated with melatonin (M) at different concentrations. After 1 h, 24 h, and 48 h culture, 0.5 mM H_2_O_2_ (H) was added and cultured for 24 h. Cells cultured with H_2_O_2_ alone were used as the controls. Cell viability was evaluated by the microculture tetrazoline test and expressed as percentages of controls (mean±SD in triplicate tests). Error bars represent SD **A**: Pretreatment with low concentrations of melatonin at 10^−10^ M (M-10) for 1 h, 24 h, and 48 h significantly protected cells against H_2_O_2_. **B**: Pretreatment with high concentrations of melatonin at 10^−6^ M (M-6) obtained similar results. The difference between cells cultured with and without melatonin at both high and low concentrations was statistically significant at all three different pretreatment periods. *p<0.05, compared with the controls.

Melatonin at concentrations of 10^−10^ M to 10^−4^ M applied for different time periods did not influence the cell viability of cultured human RPE cells (ARPE-19 cell line). Pretreatment of RPE cells with melatonin at low (10^−10^ M) or high (10^−6^ M) concentrations for 1 h, 24 h, and 48 h significantly protected cells against H_2_O_2_ damage (Figure 3A,B). Cell viability for cultures pretreated with 10^−10^ M melatonin for 1 h, 24 h, and 48 h was, respectively, 125%, 133%, and 121% of that of cells treated with H_2_O_2_ alone. Differences between cells cultured with and without melatonin pretreatment were statistically significant (p<0.05 for all three different pretreatment periods; see Figure 3A). No significant difference (p>0.05) in cell viability could be detected between cells pretreated with melatonin at 1 h, 24 h, and 48 h before challenge (p>0.05). Pretreatment with 10^−6^ M melatonin for different periods also significantly protected cells (p<0.05 in all groups; Figure 3A). No difference in cell viability could be detected between cells pretreated with melatonin for different time periods (p>0.05). Therefore, pretreatment of melatonin for 24 h was selected for subsequent experiments. Studies of the primary-culture human RPE cells from the donors obtained similar results (data not shown).

**Figure 4:**
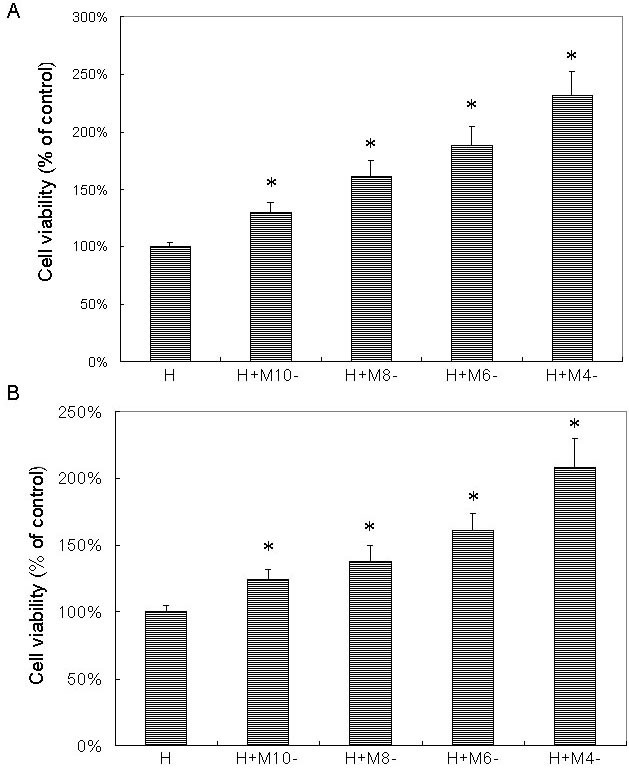
Melatonin dose-dependently protected retinal pigment epithelial cells against H_2_O_2_ damage as tested by microculture tetrazoline test. Retinal pigment epithelial (RPE) cells were pretreated with melatonin (M) at concentrations of 10^−10^ M (M-10), 10^−8^ M (M-8), 10^−6^ M (M-6), and 10^−4^ M (M-4). After 24 h, 0.5 mM H_2_O_2_ (H) was added and cultured for 24 h. Cells treated with H_2_O_2_ alone were used as the controls (H). Cell viability was evaluated by the microculture tetrazoline test and expressed as percentages of the controls (mean±standard deviation [SD] in triplicate tests). Error bars represent SD. Pretreatment with melatonin showed dose-dependent protective effects on ARPE-19 cells (an immortal RPE cell line from a 19-year-old donor) against H_2_O_2_ damage (**A**). Studies in primary culture of human RPE cells isolated from the donor eye showed similar results (**B**). *p<0.05, compared with the controls (cells treated with H_2_O_2_ alone).

**Figure 5:**
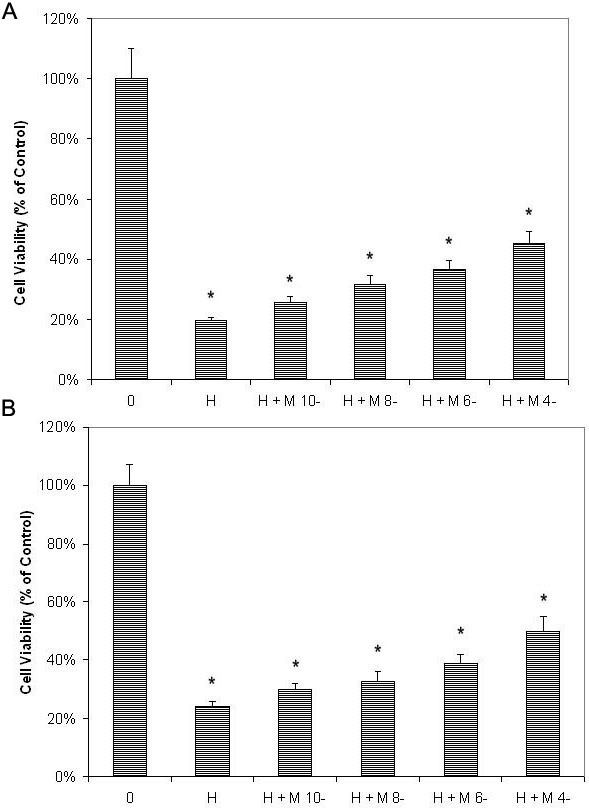
Melatonin dose-dependently protected retinal pigment epithelial cells against H_2_O_2_ damage as tested by microculture tetrazoline test, compared with cells not treated with H_2_O_2_. Retinal pigment epithelial (RPE) cells were pretreated with melatonin (M) at concentrations of 10^−10^ M (M-10), 10^−8^ M (M-8), 10^−6^ M (M-6), and 10^−4^ M (M-4). After 24 h, 0.5 mM H_2_O_2_ (H) was added and cultured for 24 h. Cells not treated with H_2_O_2_ were used as negative controls (0). Cell viability was evaluated by the microculture tetrazoline test and expressed as percentages of negative controls (mean±standard deviation [SD] in triplicate tests). Error bars represent SD **A**: Cell viability of cell treated with melatonin and H_2_O_2_ still significantly lower than that in cells cultured without H_2_O_2_ in the ARPE-19 cells (an immortal RPE cell line from a 19-year-old donor). **B**: The same was true in the primary-culture RPE cells. * p<0.05, compared with the negative controls (cells treated without H_2_O_2_).

Pretreatment of melatonin at different concentrations for 24 h showed dose-dependent protective effects on RPE cells (ARPE-19) against H_2_O_2_ damage (Figure 1 and Figure 4A). Cell viability of RPE cells pretreated with 10^−10^ M, 10^−8^ M, 10^−6^ M, and 10^−4^ M melatonin was 130%, 160%, 187% and 230%, respectively, of cells treated with H_2_O_2_ alone. The differences in cell viability between cells treated with and without melatonin were significant (p<0.05) in all groups. Using cells cultured without H_2_O_2_ as the control, the cell viability of cells treated with H_2_O_2_ alone and that of cells treated with H_2_O_2_ after pretreatment with 0 M, 10^−10^ M, 10^−8^ M, 10^−6^ M, and 10^−4^ M melatonin was 20%, 26%, 31%, 37%, and 45% of the controls, respectively, which was significantly lower than the cell viability of cells cultured without H_2_O_2_ (one-way ANOVA, p>0.05; see Figure 5A). Studies of primary-culture human RPE isolated from the donor eye showed similar results (Figure 1, Figure 4B, and Figure 5B).

### Influence of luzindole on the protective effects of melatonin on retinal pigment epithelium cells

Cell viability of cultures treated with luzindole at 50 μM alone or luzindole with melatonin at various concentrations (10^−10^-10^−4^ M) did not show any effect in either of the RPE cell lines tested.

**Figure 6:**
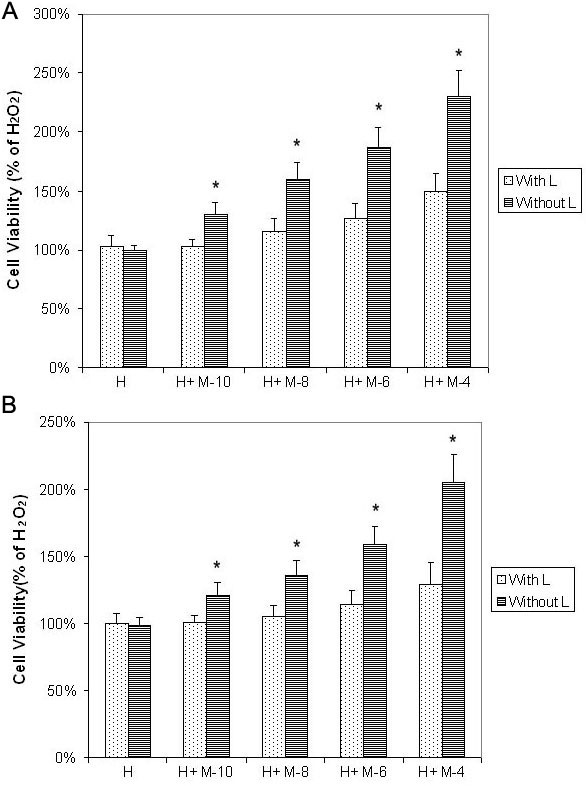
Luzindole decreased protective effects of melatonin on retinal pigment epithelial cells against H_2_O_2_ damage as tested by microculture tetrazoline test. Cultured retinal pigment epithelial (RPE) cells were treated with or without 50 μM luzindole (L). One hour later melatonin (M) was added to the culture medium at concentrations of 10^−10^ M (M-10), 10^−8^ M (M-8), 10^−6^ M (M-6), and 10^−4^ M (M-4). After 24 h, 0.5 mM H_2_O_2_ (H) was added and cultures were incubated for 24 h. Cell viability was evaluated by the microculture tetrazoline test and expressed as percentages of cells cultured with H_2_O_2_ alone (mean±standard deviation [SD] in triplicate tests). Error bars represent SD **A**: Luzindole significantly decreased melatonin-induced protective effects at 10^−10^ to 10^−4^ M in ARPE-19 cells (an immortal RPE cell line from a 19-year-old donor). **B**: The same was true in the primary-culture RPE cells. * p<0.05, comparison between cells treated with and without luzindole.

Cell viability of ARPE-19 cells (cultured with H_2_O_2_) with luzindole before melatonin was significantly decreased, compared with cells treated with melatonin at all concentrations (Figure 1 and Figure 6A). The difference in cell viability between cells treated with and without luzindole was statistically significant (p<0.05) in cells treated with 10^−10^ M to 10^−4^ M melatonin.

In cells pretreated with melatonin, the difference between cell cultures treated with H_2_O_2_ alone and cell cultures treated with H_2_O_2_ plus melatonin and luzindole was statistically nonsignificant (p>0.05) in melatonin at 10^−10^ M and 10^−8^ M (melatonin) and significant (p<0.05) in melatonin at 10^−6^ M and 10^−4^ M, by one-way ANOVA. Studies of primary-culture human RPE cells isolated from the donor eye showed similar results (Figure 1 and Figure 6B).

## Discussion

H_2_O_2_ is a relatively weak oxidant, but in the presence of metal catalysts, it can convert to the reactive hydroxyl radical, which is cytotoxic. H_2_O_2_ is the most stable ROS; it presents in tissues with a relatively long half-life. H_2_O_2_ is soluble in both lipid and aqueous media, so it can easily diffuse in and out of the cell to reach targets. For these reasons, H_2_O_2_ exposure is one of the most common in vitro models used for evaluating oxidative-stress damage on cells [10-22,26].

Previous studies have demonstrated that melatonin protects various cells against H_2_O_2_ damage in vitro, and is cell type-specific [10-21]. Little is known about the protective effects of melatonin on RPE cells against H_2_O_2_ damage. Only one paper has been published on this subject [22], which tested the effects of melatonin at very high concentrations; and even the minimum concentration tested (10^−7^ M) is 100 to 500 fold the melatonin levels in the serum. The mechanism of the protective effects (whether a direct antioxidant effect or an indirect effect through the activation of melatonin receptors) has not been studied [22].

The present study tested the protective effects of melatonin on RPE cells against H_2_O_2_ across a wide range of concentrations. Melatonin showed dose-dependent protective effects from 10^−10^ M to 10^−4^ M. The protective effects present at both physiologic concentrations (10^−10^ M to 10^−8^ M, which are 1/10 to 10 fold the melatonin serum levels) and pharmacological concentrations (10^−6^ M to 10^−4^ M). Cell survival increased by 30%–130% in cells exposed to H_2_O_2_ following melatonin pretreatment, compared to those exposed to H_2_O_2_ alone, but the cell viability in cells treated with melatonin and H_2_O_2_ was still lower than that in cells cultured without H_2_O_2_. This suggests that melatonin can partially inhibit the cytotoxicity of H_2_O_2_ on cultured human RPE cells and is consistent with previous studies demonstrating the protective effects of melatonin on other cells (neurons, astrocytes, pituitary cells, neuroblastoma cells, etc.) exposed to H_2_O_2_ [10-21]_._

Normal melatonin levels in the serum during the night have been reported to vary from 2 × 10^−10^ M to 10^−9^ M [27-29], and the melatonin levels in ocular aqueous humors have been reported to range from 10^−8^-10^−9^ M [30,31]. Urinary aMT6s levels in AMD patients were 40% lower than in those of age- and gender-matched controls [8]. The present study suggests that melatonin at 10^−8^-10^−10^ M significantly protects human RPE cells against H_2_O_2_ in a dose-dependent manner. It corroborates the hypothesis that the deficiency of melatonin in AMD patients may play a role in the pathogenesis of AMD and that supplementation of melatonin may be helpful in the prevention and treatment of AMD.

Melatonin may protect cells against oxidative damage by at least two mechanisms. At high concentrations, melatonin may act as a scavenger of free radicals, ROS, and reactive nitrogen species. The redox properties of melatonin are similar to those of other tryptophan metabolites [32]: melatonin quenches free radicals (superoxide and hydroxyl radicals) efficiently but quenches singlet oxygen with only moderate efficiency [33]. On the other hand, it is able to activate membrane-bound melatonin receptors, which can stimulate the production of a variety of antioxidative enzymes through several signaling pathways [21,27,34-40].

To test whether the protective effects of melatonin against H_2_O_2_-induced oxidative stress demonstrated here were due to direct ROS quenching or due to indirect receptor-mediated effects, we employed luzindole, a melatonin receptor antagonist. Luzindole is a nonselective antagonist of melatonin membrane receptors MT_1_ and MT_2_, with a higher affinity for the MT_2_ subtype. It has been extensively used to distinguish the direct antioxidant effects from indirect receptor-mediated effects [35,41-47].

Very little is known about the effect of luzindole on melatonin-induced protection of cells against H_2_O_2_ damage. Only one report mentions that luzindole attenuated the effects of melatonin-induced protection of motoneurons against H_2_O_2_ damage, at a single melatonin concentration of 1.5×10^7^ M [21].

Human RPE cells express MT2 but not MT1 melatonin membrane receptors [30,48]. Luzindole significantly decreased the melatonin-induced protective effects at all concentrations of melatonin, indicating that the receptor-dependent indirect effects are the main protective mechanism in RPE cells. This is consistent with studies performed on motoneurons at a single concentration [21]. Binding of melatonin with its membrane receptors appears to promote enzymes that metabolize oxidative stress. These enzymes include glutathione peroxidase, glutathione reductase, superoxide dismutases, glucose-6-phosphate dehydrogenase, and catalase [36]. Additionally, melatonin receptors can couple to multiple signaling pathways, which protects cells from oxidative stress damage [21].

In the present study, pretreatment of RPE cells with luzindole decreased but did not completely block the protective effects of melatonin at high concentrations (10^−6^-10^−4^ M), suggesting that at pharmacological concentrations, melatonin may have direct antioxidant effects. This indicates that luzindole, by blocking the activation of melatonin membrane receptors, can completely abolish the protective effect of melatonin at low concentrations. However, luzindole can reduce, but cannot completely abolish, the protective effect of high levels of melatonin against H_2_O_2_ damage.

Controversy exists as to whether melatonin can neutralize H_2_O_2_ directly [36,38]. However, H_2_O_2_ can convert to the hydroxyl radical, a highly reactive species, which is very toxic to cells and is the main cause of cell damage from H_2_O_2._ The hydroxyl radical is not enzymatically detoxified within cells and can only be neutralized by direct free-radical scavengers. Melatonin interacts with hydroxyl radicals at a rate-constant that is equivalent to that of other highly efficient hydroxyl radical scavengers [27,32,34,35,38]. Therefore, this could be the mechanism for receptor-independent melatonin protection.

In addition to the protective effects of melatonin on human RPE cells against oxidative stress, previous studies have also demonstrated that melatonin protects retinal homogenates against lipid peroxidation [49], and protects photoreceptors against various oxidative stressors [50-52]. These studies have also suggested that melatonin may be beneficial in the management of AMD.

Only one clinical study explored the treatment of AMD with melatonin supplementation: Yi et al. [53] reported encouraging results, but the study was nonrandomized, with only 6 months of follow-up and an attrition rate at follow-up of 45%. Still, these results are encouraging for organizing a randomized clinical trial evaluating the therapeutic value of melatonin supplementation for AMD management.

Supplementation of melatonin has been widely used in the treatment of various human disorders. The doses range from 1 mg to 5 mg per night (low dosage) for treating insomnia or jet lag [27,54] to 20 mg or more per day (high dosage) for managing malignant tumors [55]. Patients taking a low dosage at night usually have no significant side effects, whereas sleepiness, fatigue, seizures, or mild nausea may occur in patients taking a high dosage [55,56]. If melatonin protects cells against oxidative stress only at pharmacological concentrations (10^−6^-10^−4^ M), then a high dosage would be required (e.g., an orally administered dose of 80 mg melatonin raises melatonin serum levels from 10^−7^ M to 2×10^−6^ M) [57]. Our study suggests that melatonin effectively protects RPE cells at physiologic levels (10^−8^-10^−10^ M), which can be obtained from an orally administered, low dosage of melatonin (1–5 mg per night; e.g., an orally administered dose of 3 mg melatonin has been reported to raise the melatonin serum level to 2×10^−8^ M) [28]. This suggests that oral administration of 1–3 mg melatonin per night should be sufficient to test the clinical efficacy of melatonin therapy in AMD patients.
